# Wound construction in manual small incision cataract surgery

**DOI:** 10.4103/0301-4738.58486

**Published:** 2010

**Authors:** Rupesh Agrawal

**Affiliations:** Ocular trauma, Uveitis and Comprehensive Ophthalmology, L V Prasad Eye Institute, Hyderabad, India

Dear Editor,

I read the article on wound construction in manual small incision cataract surgery (MSICS) by Haldipurkar *et al*.[1] The authors need to be complimented for their candid expositions on scleral wound construction in widely done SICS. I will like to add a few comments on scleral wound construction in MSICS:

- Status of the sclera: It is imperative to look at the status of sclera before one plans for scleral wound construction. Cases with thin sclera such as high myopia, osteogenesis imperfecta, Ehler Danlos Syndrome, healed scleritis are relative contraindications for scleral wound construction.
- Incision with 15 No. Blade: Surgeon needs to make the scleral bed dry with the help of cotton bud as making the incision on a relatively wet scleral bed can lead to splaying of the scleral fibers leading to irregular astigmatism.
- Placement of the crescent knife: The horizontal shaft of the crescent knife needs to be touching close to the sclera (sclera posterior to wound) while dissecting the scleral bed with the knife as it will lead to minimum complications; if it is not abutting close to sclera and if the horizontal shaft is away from posterior sclera it can lead to premature entry.
- Formation of the anterior chamber with viscoelastic agent: Surgeon needs to form the anterior chamber with viscoelastic agent or to switch on the anterior chamber maintainer if one is doing MSICS by Blumenthal technique, in order to achieve smooth dissection of scleral bed. Hypotonus globe can act as determinant for smooth tunnel construction. At the same time, one needs to be careful not to overfill the anterior chamber as it can lead to premature entry.
- Author mentions that the crescent blade should be cutting while being brought out of the tissue,[1] but there is no mention about the cutting of sclera with keratome. All the cutting needs to be done while the keratome is moving forward into the scleral tissue in order to prevent irregular astigmatism.
- In cases with premature entry or with leak from the tunnel the suturing needs to be done by using ‘figure of 8’ or ‘infiniti’ suture.

Incorporation of the above intraoperative surgical tips into one‘s surgical armamentarium will ensure a safer and smooth MSICS for the novice surgeon.
